# Knotty inflation and the dimensionality of spacetime

**DOI:** 10.1140/epjc/s10052-017-5253-3

**Published:** 2017-10-14

**Authors:** Arjun Berera, Roman V. Buniy, Thomas W. Kephart, Heinrich Päs, João G. Rosa

**Affiliations:** 1 Tait Institute, School of Physics and Astronomy, University of Edinburgh, Edinburgh, EH9 3FD UK; 2 Schmid College of Science, Chapman University, Orange, CA 92866 USA; 3 Department of Physics and Astronomy, Vanderbilt University, Nashville, TN 37235 USA; 4 Fakultät für Physik, Technische Universität Dortmund, 44221 Dortmund, Germany; 5 Departamento de Física da Universidade de Aveiro and CIDMA, Campus de Santiago, 3810-183 Aveiro, Portugal

## Abstract

We suggest a structure for the vacuum comprised of a network of tightly knotted/linked flux tubes formed in a QCD-like cosmological phase transition and show that such a network can drive cosmological inflation. As the network can be topologically stable only in three space dimensions, this scenario provides a dynamical explanation for the existence of exactly three large spatial dimensions in our Universe.

## Introduction

Although the question of why our Universe has exactly three (large) spatial dimensions is one of the most profound puzzles in cosmology – especially in view of quantum gravity scenarios such as string theory which assume nine or ten space dimensions at the fundamental level – it is actually only occasionally addressed in the literature [1–22]. In this paper we propose a topological explanation for the dimensionality of space-time based on the idea that inflation is driven by a tightly knotted network of flux tubes generated in a cosmological phase transition and the fact that knots are topologically stable only in exactly three space dimensions (“knotty inflation”).

The main idea of the model is connected to the fact that, in non-Abelian gauge theories analogous to QCD, chromoelectric flux tends to become confined and the resulting tube-like structures can be treated as effective one-dimensional objects or strings. The formation of flux tubes is behind the basic models of hadronization in QCD, with flux strings connecting quark–antiquark pairs giving rise to a linear potential that confines them into mesons and prevents the existence of free colored charges. A similar description with more complicated flux tube shapes also describes the formation of baryons and glueballs, the latter denoting bound states of pure gauge fields.

During the phase transition from a generic quark–gluon plasma to a hadron gas, a large number of flux tubes may fill up the whole Universe and form an intricate network of both open and closed flux tubes, with some similarities to what is known as the ‘spaghetti vacuum’ [23–27], potentially with a large linking or crossing number density provided the flux tube density is sufficiently large. A related idea has been suggested recently in [28]. It has been shown that such a network is stabilized by a topological conservation law [29, 30].

This network will tend to relax into a tight configuration that fills the whole space such that each tube minimizes its energy, similarly to the description of glueballs in terms of knotted/linked flux tubes proposed in [31–33]. The network will then decouple from the Hubble flow.

As a result, the energy stored in this tight network will provide an effective cosmological constant which is homogeneous on scales large compared to the typical flux tube width and on average isotropic if the flux tubes are randomly oriented. This effective cosmological constant will thus trigger a period of inflation in the early Universe that lasts until the network decays through reconnection, tube breaking or other quantum effects. Network breaking will proceed until a gas of hadrons and radiation is formed and the standard cosmological evolution begins.

Of all possible dimensionalities of space, our mechanism picks out three as the only number of dimensions that can inflate and thus become large. String theory and various types of Kaluza–Klein theories are explicit examples where different spacetime dimensionalities are possible. For example, in string theory, which is an intrinsically higher-dimensional theory, gauge theories are confined to hypersurfaces of *p* spatial dimensions known as D*p*-branes, to which open strings are attached. This concept has been employed in a wide range of extra-dimensional scenarios, where fields are confined to lower-dimensional slices of a higher-dimensional spacetime.

The main difference between the dynamics of confinement in $3+1$ and higher-dimensional gauge theories lies in the fact that flux tube knots and links will only be topologically stable (or metastable) in three spatial dimensions, being otherwise able to unknot/unlink in the extra dimensions. Given that this is an essential feature for the formation of a tight network of flux that decouples from the Hubble flow and provides an effective cosmological constant, confinement can only lead to inflation for gauge theories living in three-dimensional hyperspaces.

The argument can be used in two different scenarios: First, in string theory, particles represented as open strings are attached to *n*-dimensional hypersurfaces (“branes”). Only particles confined to 3-branes will produce a topologically stable network, and only these branes will inflate. (Note that in such a case an additional mechanism to stabilize the extra dimensions is nevertheless required, although this is beyond the scope of the present work.) Alternatively, the entire Universe could have *n* dimensions. In this case of all possible Universes only Universes with three dimensions will be able to inflate. Thus this scenario can explain why our Universe has only three large spatial dimensions in the absence of other sources of vacuum energy.

It is the purpose of this Letter to outline the main features of this mechanism and discuss the necessary properties that a flux tube network must exhibit in order to yield a successful inflationary model in three dimensions. While leaving a detailed modeling of the network’s evolution and observational predictions of this scenario for a future work, we will describe the most important physical processes that drive the network’s formation, inflationary dynamics and subsequent decay, giving quantitative estimates for the relevant time and energy scales.

## Flux tubes in gauge theories

Understanding confinement in QCD and gauge theories in general is one of the most important problems in particle physics. Due to the strong coupling and non-perturbative nature of confinement, this is an intrinsically hard problem that can currently only be studied accurately in the context of numerical simulations on the lattice. It is nevertheless widely accepted that the confinement of chromoelectric charges, such as quarks and anti-quarks in QCD, is associated with a squeezing of chromoelectric flux lines into tube-like structures connecting the charges. These flux tubes then give rise to a potential energy that grows linearly with the distance between pairs of quarks and anti-quarks, therefore leading to their confinement in mesons, baryons and potentially other hadronic structures. Flux tubes can be described e.g. in terms of Abrikosov–Nielsen–Olesen vortices [34, 35] in dual superconductor models of confinement (see [36] for a review).

It was pointed out in [31–33] that closed flux tubes may have non-trivial topologies, including knotted tubes and links between distinct closed flux tubes. The degree of “knottedness” of each configuration is then associated with a conserved topological charge, analogous e.g. to the (Abelian) magnetic helicity that characterizes conducting fluids in magnetohydrodynamics [37, 38]. Any such topologically non-trivial flux tube configuration will necessarily relax into an equilibrium state by minimizing its length and therefore its energy. These equilibrium states correspond to the tightest knotted or linked configurations with a given topological charge and number of flux quanta. This thus motivates us to consider the description employed in [31–33], where the chromoelectric field $F_{0i}$ (where $i=1,2,3$ in three spatial dimensions) is confined in knotted/linked tube-like structures that carry one quantum of chromoelectric flux.

Just like in standard Big Bang cosmology, where it is difficult to generate seed magnetic fields, here we also assume that our QCD analog gauge theory does not generate magnetic fields. We assume there are particles with analog chromoelectric charges, but no analog chromomagnetic monopoles. One could include this generalization, but it would unduly complicate the analysis.

The relevant (static) Lagrangian density is given by

1 $$\begin{aligned} \mathcal {L}={1\over 2}\mathrm {tr}\left[ F_{0i} F^{0i}\right] +\mathrm {tr}\lambda \left[ {\Phi _\mathrm{E}\over (\pi a^2)}- n^iF_{0i}\right] , \end{aligned}$$

up to the addition of a constant energy density as in the MIT bag model [39, 40], where the last term enforces flux conservation across the tube sections through a Lagrange multiplier $\lambda$, with $n^i$ denoting the unit vector normal to the tube’s cross section. We expect the flux tube radius *a* to be given by the confinement scale $\Lambda$, such that $a\sim \Lambda ^{-1}$ in natural units, which we will employ in our discussion henceforth. This yields the equations of motion for the gauge field:

2 $$\begin{aligned} D^0 (F_{0i}-\lambda n_i)=0 , \qquad D^{i}(F_{0i}-\lambda n_i)=0 , \end{aligned}$$

the solution of which is given by a constant chromoelectric field

3 $$\begin{aligned} F_{0i}={\Phi _\mathrm{E}\over \pi a^2}n_i, \end{aligned}$$

which vanishes outside the flux tube. The energy density inside each flux tube is then given by

4 $$\begin{aligned} \rho _E={1\over 2}{\mathrm {tr}\Phi _\mathrm{E}^2\over (\pi a^2)^2}\sim {\Lambda ^4\over 2\pi ^2} , \end{aligned}$$

up to $\mathcal {O}(1)$ factors that will not affect our discussion.

## Network formation

Let us then hypothesize that there exists a confining gauge theory, analogous to QCD but with a generic high-energy confinement scale $\Lambda$, and which lives on a three-dimensional brane within a higher-dimensional compact space. For simplicity, we will denote the particles charged under this gauge group as quarks and gluons, although one must bear in mind that these are not the known QCD fields but rather novel degrees of freedom that must decouple from the low-energy effective theory.

In the early Universe, for temperatures above a certain critical value $T_\mathrm{c}\sim \Lambda$, these quarks and gluons are essentially free, forming a plasma that we will assume is close to thermal equilibrium and dominates the energy balance in the Universe. This plasma has an energy density $\rho _\mathrm{R} (T)=(\pi ^2/30) g_* T^4$, where $g_*$ denotes the number of relativistic degrees of freedom, which includes at least the massless gluons and the light Standard Model fields of the theory. Due to expansion, the temperature of the plasma will decrease until it becomes lower than the critical value, at which point all the free gluons become confined in both open and closed flux tubes, the former connecting quark–antiquark pairs. Given that

5 $$\begin{aligned} \rho _\mathrm{R} (T_\mathrm{c})\sim g_*\Lambda ^4 \sim \rho _\mathrm{E}, \end{aligned}$$

we expect that, at the confining phase transition, a large number of flux tubes is formed within each Hubble volume. Such a large density will naturally lead to a large number of crossings between the flux tubes and, hence, to a large number of knots and links of different configurations. This is analogous to the behavior observed when a string is tumbled inside a box of fixed volume, where the probability of knotting increases very quickly above a certain critical string length and which effectively corresponds to a critical density [41]. The flux tube network will then be endowed with a non-trivial topology, which can be metastable in three spatial dimensions and that, as we describe below, can be sufficiently long-lived to drive a period of inflation in the early Universe.

## Inflation

After the phase transition, Hubble expansion will tend to stretch the flux tubes, while maintaining their radius (set by the strong non-perturbative dynamics), and increase the size of the gaps in between them. However, as argued above, the presence of knots and links makes the network behave differently from a system of isolated flux tubes, endowing it with a rigidity and making it try to relax into a tight equilibrium configuration. Flux tubes will then shrink and approach each other in order to maximize the fraction of the spatial volume that they can occupy given the topological constraints.

The number of knots and links in the network will decrease due to the decay processes that we describe below. These will make the network progressively less tight, with the tightest configuration occupying an increasingly smaller fraction of the spatial volume.

We then expect a network to remain tight if

6 $$\begin{aligned} \tau _{\text {tight}} \ll H^{-1} \ll \tau _{\text {decay}} , \end{aligned}$$

i.e. if it relaxes into a tight configuration more quickly than expansion and knots/links are stable on the Hubble time scale. If the network is sufficiently dense at the phase transition, i.e. if the gaps between flux tubes are not much wider than the tube radius, we expect $\tau _{\text {tight}}\lesssim \Lambda ^{-1}$, and the network can in principle relax into a tight configuration within a Hubble time for $\Lambda \gg H$. In this case, the tube width is also much smaller than the Hubble radius during inflation, so that the network is essentially homogeneous on near-horizon and super-horizon scales. It should also be, on average, isotropic if quarks and gluons are randomly distributed in the thermal plasma at the transition. Since the background inflationary dynamics is dictated by the near- and super-horizon properties of the dominant fluid, we may consider a homogeneous and isotropic expansion as a first approximation. The underlying anisotropic structure of the knotted flux tube network may nevertheless give rise to small deviations from isotropy that could e.g. be analyzed within the framework developed in [42]. One should also note that, as long as the gaps in the network never attain super-Hubble sizes, the process of tightening can occur at sub-luminal (and even non-relativistic) flux tube speeds, being consistent with causality.

Let us see that a tight network will behave as an effective cosmological constant as long as the above time scale hierarchy is satisfied. The “tightness” of the network can be evaluated in terms of the fraction of the spatial volume occupied by the flux tubes, *f*, which will in general depend on the space and time coordinates. On average, we expect a network that is homogeneous and isotropic on scales larger than the tube radius, such that $f=f(t)$ at leading order. The network thus has an average energy density $\rho =f \rho _\mathrm{E}$, with the first law of thermodynamics yielding

7 $$\begin{aligned} \mathrm{d}(\rho V)= {\mathrm{d}f\over \mathrm{d}V}(\rho _\mathrm{E} V) \mathrm{d}V+f\rho _\mathrm{E} \mathrm{d}V= -p\mathrm{d}V , \end{aligned}$$

where we neglected decay processes and consequent heat transfer. From this we conclude that the network has an effective pressure:

8 $$\begin{aligned} p=-\left( 1+{1\over 3}{\mathrm{d}\log f\over \mathrm{d}N_\mathrm{e}}\right) \rho , \end{aligned}$$

where $N_\mathrm{e}$ denotes the number of e-folds of expansion, and hence we have an equation of state parameter $w=p/\rho \simeq -1$ for $\dot{f}/f \ll 3H$. Note that, as discussed above, we expect the network to be dense at the phase transition and hence close to a tight configuration that fills a significant fraction of the spatial volume, with $f\lesssim 1$.

The network will initially relax into a tight configuration with $\mathrm{d}\log f/\mathrm{d}N_\mathrm{e} > 0$, yielding a phantom-like equation of state $w<-1$ [43]. Although this phantom behavior often leads to instabilities [44, 45], it is nevertheless considered in dynamical dark-energy models, as well as in dark-matter scenarios with bulk viscosity [46], and it would be interesting to consider this behavior in the context e.g. of axionic strings [47].

The network will then subsequently remain close to a tight configuration, with a fixed or at most slowly varying filling fraction *f*. The rigidity of the network, which results from the presence of a large density of knots and links, will thus decouple it from the Hubble flow and generate an effective cosmological constant.

As the number of knots and links decreases due to network decay, *f* will then decrease such that $w\gtrsim -1$, thus yielding a period of inflation with an equation of state analogous to that of a canonical slowly rolling scalar field. Note that, in the absence of the topological charge that maintains the tightness of the network, the filling fraction would decrease quickly and no accelerated expansion could be obtained.

To better understand this, note that, in the absence of a non-trivial topology, a network of long non-relativistic tubes has an equation of state $p = -\frac{1}{3}\rho$, as a result of the fact that only the tube length increases with expansion, while its cross-section remains fixed due to flux conservation. On the other hand, a two-dimensional network of small string loops (or also e.g. a two-dimensional braid of long tubes) at rest will behave, up to small distortions, as a rubber sheet or domain wall, with an equation of state $p=-\frac{2}{3}\rho$. If we then add a stack of two-dimensional chain mails and link them together to form three-dimensional space filling chain mail, we get $p = - \rho$, as long as the network is stable and the network density remains constant.

For completeness it should be mentioned that the expansion law for the flux tubes could also be modified to $p = -(1/3 + \beta ) \rho$ for some nonzero $\beta$ by conformal symmetry breaking, similar to treatments in cosmic *B* fields.

Notice that the number of knots in a comoving volume is not constant in this scenario. As the Universe expands, the gaps in the network would tend to expand in the absence of knots or links, which would dilute the network. Knotting prevents this dilution by keeping the gaps between neighboring tubes at an approximately fixed size, thus maintaining the average energy density. In order to sustain inflation in such a scenario an inflating region necessarily has to get filled with more and more flux tubes as it expands. Again, there are two possible realizations how this could happen: flux tubes could either be pulled into the inflating patch from neighboring regions or produced inside the patch itself.

In the first case, if the three dimensions where the knotted network lives are infinite in extent, this would allow inflation to occur everywhere, since there would be an infinite supply of flux tubes that may relax into a tight configuration. On the other hand, for compact three dimensions the network is necessarily finite and inflation will only occur in the regions that tighten faster and, in the process, pull in flux tubes from neighboring (non-inflating) regions. This may pose constraints on the initial size of our three dimensions such that at least the small patch that later became the presently observable Universe has inflated. Note that it is not mandatory that the flux tubes from outside the patch completely get pulled inside the initial inflationary patch. If they only get partially pulled in, it will just mean that the regions around the initial patch then join the initial patch in inflating. The key point is once there is an overdensity of flux tubes that initiate inflation, they act as an attractor for more flux tubes.

In the above picture, flux tubes are seen as (semi)classical objects that arise from the confinement of gluons in the thermal bath after the phase transition and there is no mechanism that, in this case, can create or destroy flux tubes afterwards. Flux tube production is, however, not prohibited by any conservation law, and the topologically metastable network could be seen as a “false vacuum” configuration of the chromoelectric field after the phase transition. In this case, expansion does not preserve the number of flux tubes in a comoving volume but rather the false vacuum field structure, which contains the knotted network of tube-like field configurations.

Just as in scalar field models of inflation, in this case gravitational energy is converted into vacuum energy with the same field structure as more space is created by expansion, as for the vacuum-energy description of the present-day cosmological constant. Although more speculative in nature, this alternative description would allow a single patch to inflate independently of the initial extension of the flux tube network, being in this sense more attractive.

In both of the above scenarios, inflation can only occur in three dimensions and lead to a uniform Universe with three large dimensions at late times. The Friedmann equation yields

9 $$\begin{aligned} H^2\simeq {f \Lambda ^4\over 6\pi ^2 M_\mathrm{P}^2} \end{aligned}$$

during inflation, which implies that $H<\Lambda$ for a sub-Planckian confinement scale, with e.g a phase transition at the grand unification scale, $\Lambda \sim 10^{16}$ GeV, yielding $H/\Lambda \sim 10^{-3}$ for $f\lesssim 1$. This shows that the flux tube network will in general be on average homogeneous and isotropic on super-horizon scales, since the tube radius $a\sim \Lambda ^{-1} \ll H^{-1}\sim \sqrt{6\pi ^2/ f} (M_\mathrm{P}/\Lambda )\Lambda ^{-1}$, and that relaxation may in principle occur in less than a Hubble time, as required above.

## Network decay and reheating

A knot or link between two flux tubes is only classically stable if these are unable to intersect and either reconnect or pass through each other. Such intercommutations lead to the well-known scaling behavior in cosmic string networks, which has been observed in several examples of non-interacting strings (see e.g. [48, 49]). Adjacent tubes may, however, repel each other as a result of the surface currents that support their flux. This has been observed in type II superconductors, where repulsion leads to the formation of hexagonal lattices as first predicted by Abrikosov (see [50] for a review). Evidence for a repulsive interaction between non-Abelian flux tubes has also been found in the SU(2) Yang–Mills theory [51].

Flux tubes with small velocities will be classically forbidden from overcoming this repulsive barrier and thus from intersecting and reconnecting. This is e.g. supported by two-dimensional simulations of vortex collisions, where it has been shown that colliding vortices only overlap above a critical velocity [52]. We expect typical velocities to be indeed small in our scenario due to the large number density of mutually repelling flux tubes formed during the phase transition, as discussed above. We note that relativistic tubes can overcome the repulsive barrier and reconnect [53], but this is not the relevant regime for the present scenario.

Although classically forbidden, reconnection may nevertheless occur through quantum processes similar to those responsible for the metastability of knotted/linked glueballs in [31–33].

Two flux tubes may unknot/unlink by quantum tunneling. We can estimate this by treating the intersection between two flux tubes as a non-relativistic particle of mass $m\sim \rho _\mathrm{E} a^3\sim \Lambda /2\pi ^2$ that tunnels through a potential barrier of length $\sim a$ and height $\Delta V\gtrsim \Lambda$. This yields a typical lifetime in units of the Hubble time:

10 $$\begin{aligned} \tau _t H={C\over v}(aH) \exp \left( {2D\over \pi } \sqrt{\Delta V\over \Lambda }\right) , \end{aligned}$$

where $v\ll 1$ is the typical tube velocity and *C*, *D* are $\mathcal {O}(1)$ factors parametrizing the estimate’s uncertainty.

Flux tubes can also break through the well-known Schwinger effect [54], where $Q\bar{Q}$ pairs are produced in the approximately constant chromoelectric field enclosed within each flux tube. For $N_\mathrm{f}$ quark flavors of mass $m_Q$, using the fact that the string tension $\kappa = \pi a^2\rho _\mathrm{E}\sim \Lambda ^2/(2\pi )$, we obtain for the lifetime of a flux tube of length *l*

11 $$\begin{aligned} \tau _\mathrm{b} H\sim {8\pi ^4\over N_\mathrm{f}}(aH)^2(lH)^{-1} \exp \left( 2\pi ^2 {m_Q^2\over \Lambda ^2}\right) . \end{aligned}$$

This implies that the lifetime for string breaking can be parametrically large if there are no light quarks in the spectrum of the gauge theory, $m_Q\gg \Lambda$, even for $a\ll H^{-1}$ and flux tubes as long as the Hubble radius. Thus, the confining gauge theory responsible for a sufficiently long period of inflation in the proposed scenario has to be distinct from QCD, since in the latter case knots and links between flux tubes will quickly decay through the production of up and down quark–antiquark pairs.

Given the exponential suppression of the string breaking and tunneling rates, it is thus not difficult to envisage scenarios where the network remains tight for the 50–60 e-folds of accelerated expansion required to explain the present flatness and homogeneity of the Universe.

String breaking will eventually lead to a system of unknotted/unlinked flux tubes, and thus to a gas of hadrons. These may be unstable and decay into radiation, including in principle the Standard Model particles. The details of this process depend, of course, on how the confining gauge theory at a high-energy scale $\Lambda$ is related to the low-energy physics, but it is clear that the proposed model naturally includes a mechanism for reheating the Universe after inflation. It is also possible for a significant amount of radiation to be produced during the inflationary period, if e.g. small loops unlink from the network and decay while the network is still tight, thus potentially leading to a warm inflation model [55, 56].

## Cosmological perturbations

The flux tube network is, on average, homogeneous on scales larger than the tube radius, which as shown above can be parametrically below the Hubble radius. Nevertheless, fluctuations in the energy density of the flux tube network may arise on super-horizon scales and become imprinted on the curvature of space-time, thus seeding the temperature anisotropies in the Cosmic Microwave Background and the Large Scale Structure of the present Universe.

Curvature perturbations in a perfect fluid driving a period of accelerated expansion propagate with an imaginary sound speed $c_\mathrm{s}^2= \mathrm{d}p/\mathrm{d}\rho = w <-1/3$, which would make the system unstable to small fluctuations as opposed to the canonical scalar field models of inflation. However, the flux tube network has a non-vanishing rigidity as a result of the presence of numerous knots and links. The network thus behaves more like an elastic solid than a perfect fluid, similarly to the networks of topological defects considered in [57–62]. The anisotropic stress inherent to elastic solids allows for the propagation of both longitudinal and transverse waves and the associated sound speed $c_\mathrm{s}^2= w + (4/3)\mu /(\rho +p)$ can be real for a sufficiently large shear modulus $\mu$ [63].

Inflationary models with elastic solids may lead to a nearly scale-invariant spectrum of both curvature and tensor perturbations with interesting differences from scalar field models [63]. Firstly, despite the absence of non-adiabatic modes, perturbations evolve on super-horizon scales due to the presence of anisotropic stress, although maintaining the relevant scaling between different super-horizon modes. The overall amplitude of the spectrum thus depends on the details of reheating, which may potentially yield distinctive observational signatures of our model, where reheating proceeds through the decay of a knotted/linked network. Anisotropic stress will also act as a source for tensor perturbations, potentially modifying the primordial gravitational waves spectrum with respect to canonical models.

Although concrete observational predictions require a detailed modeling of the properties and dynamics of the flux tube network, which is beyond the scope of this Letter, it is worth mentioning that a red-tilted curvature spectrum can only be obtained in elastic solid models of inflation for a slowly varying equation of state. We expect this to be the case in our model since a knotted network will be close to, but not exactly in, a tight configuration, as a result of the opposing effects of Hubble expansion, relaxation and unknotting events.

## Conclusion

A knotted/linked flux tube network formed in a QCD-like phase transition can provide a natural source of inflation and is one of the few scenarios not requiring a fundamental scalar field. Furthermore, this model may explain why we live in three large spatial dimensions, since knotted/linked tubes are topologically unstable in higher-dimensional space-times. This picture may also be applied to a model of dark energy, which would eliminate the need for an ultra-light scalar field.

Although exact solutions in such models are unavailable, appropriate approximations should be enough to establish the main qualitative features. A key advantage of this model is that its underlying building block, the Abelian or non-Abelian flux tube, is a quantity that has been extensively studied and there are many tools and methodologies available to further explore it. We will present a more detailed analysis based on the Abelian Abrikosov–Nielsen–Olesen model elsewhere [64].
